# X-ray Irradiated Vaccine Confers protection against Pneumonia caused by *Pseudomonas Aeruginosa*

**DOI:** 10.1038/srep18823

**Published:** 2016-02-16

**Authors:** Yanyan Li, Zhenling Wang, Xiaoxiao Liu, Jianying Tang, Bin Peng, Yuquan Wei

**Affiliations:** 1 Department of Radiation Oncology, Fudan University Shanghai Cancer Center, Fudan University, Shanghai, China; 2 State Key Labortary of Biotherapy and Cancer Center, West China Hospital, Sichuan University, and Collaborative Innovation Center of Biotherapy, Cheng Du, China; 3 Department of Oncology, Third Xiangya Hospital, Central South University, Changsha, China

## Abstract

*Pseudomonas aeruginosa* is a gram-negative bacterium and one of the leading causes of nosocomial infection worldwide, however, no effective vaccine is currently available in the market. Here, we demonstrate that inactivation of the bacteria by X-ray irradiation inhibits its replication capability but retained antigenic expression functionally thus allowing its use as a potential vaccine. Mice immunized by this vaccine were challenged by the parental strain, the O-antigen-homologous strain PAO-1 (O2/O5) and heterologous strain PAO-6 (O6) in an acute pneumonia model. We further measured the protective effect of the vaccine, as well as host innate and cellular immunity responses. We found immunized mice could protect against both strains. Notably, the antiserum only had significant protective role against similar bacteria, while adoptive transfer of lymphocytes significantly controlled the spread of the virulent heterologous serogroup PAO-6 infection, and the protective role could be reversed by CD4 rather than CD8 antibody. We further revealed that vaccinated mice could rapidly recruit neutrophils to the airways early after intranasal challenge by PAO-6, and the irradiated vaccine was proved to be protective by the generated CD4^+^ IL-17^+^ Th17 cells. In conclusion, the generation of inactivated but metabolically active microbes is a promising strategy for safely vaccinating against *Pseudomonas aeruginosa*.

*Pseudomonas aeruginosa* (*P. aeruginosa*) *is* an opportunistic pathogen and one of the leading nosocomial infections worldwide. It is often the primary agent infecting immune compromised patients who suffer from severe burns, cancer or who is undergoing immunosuppressive therapies^1^,^2^. Despite considerable advances in the development of antimicrobial and supportive therapy, effective control or treatment strategy of *P. aeruginosa* invasion remains a persistent headache. Several studies have demonstrated the natural resistance of *P. aeruginosa* to antibiotics^3^ or its ability to evade the host’s immune system^4^,^5^. These characteristics give rise to the difficulties in treating *P. aeruginosa* infection, resulting in a desire to pursue immunotherapeutic approach to counter this persistent pathogen.

Several vaccine candidates have been investigated. They include sub-cellular antigens like structural components such as flagella, pili, outer membrane proteins or lipopolysaccharides (LPS), ormucoid exopolysaccharides (MEP), exotoxin A and proteases^6^,^7^,^8^. These vaccine candidates have been tested in phase I-III clinical trials^9^,^10^,^11^. However, despite intense efforts over the past few decades, vaccines against *P. aeruginosa* experienced little success, a safe and effective vaccine is not available currently^8^. In our approach, we intend to simultaneously target multiple antigens of P. aeruginosa with a non-replicating but metabolically active vaccine by inducing effective polyclonal antibodies.

As vaccine candidates, heat or formalin killed pathogens are safe but they usually elicit a weaker immunogenic response due to impaired antigenic structure^12^. In contrast, live vaccines may have enhanced immunogenicity and increased durability in the host, but they usually cause safety problems, particularly among the immune-compromised patients^13^. Fortunately, this dilemma in vaccine research is partly resolved by the recently developed approach of photochemical or irradiation inactivated vaccines^14^. The use of ionizing radiation has been explored in the development of vaccines for the prevention of some infectious diseases in cattle and humans the past few years^15^,^16^,^17^,^18^. This strategy allowed the completely non proliferated pathogen to present the immunologically functional epitopes, and they activate a robust immune response which is comparable to that induced by a live and unprocessed pathogen.

In this study, we demonstrate that inactivation of *P. aeruginosa* by X-ray irradiation inhibited its replication capability while retained the metabolic viability. The immunization increased survival condition of *P. aeruginosa* pneumonia suffers, and the protective role may mainly realized by T lymphocytes rather than serum antibody against heterologous serogroup infection, while both T lymphocytes and serum worked against the infection of homologous serogroup *P. aeruginosa*. The protective effect of the vaccine is further demonstrated to be mainly mediated by CD4^+^ T lymphocytes, and predominantly realized by CD4^+^ IL-17^+^ Th17 cells. These observations may provide a new vaccine preparation strategy for active immunotherapy and potential targeted therapy for *P. aeruginosa*.

## Results

### Preparation and safety evaluation of X ray irradiated vaccine

**Figure 1:**
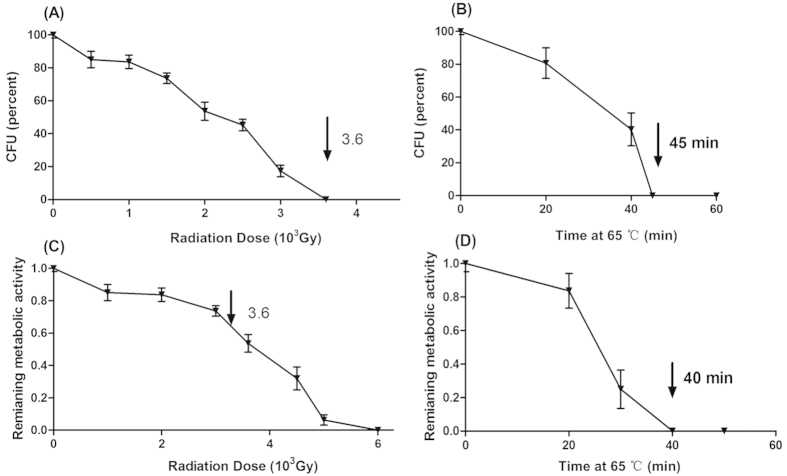
Irradiation inhibits *P. aeruginosa* reproductive viability but does not impair metabolic activity. Increased exposure time to X-ray irradiation or heat (65 °C) resulted in decreased reproductive viability (**A,B**) and metabolic activity (**C,D**) of ATCC 27853 cells. Bacterial viability was determined by counting CFUs on agar plates, and metabolic activity was measured by monitoring the OD using Alamar Blue colorimetric assay. X ray irradiation diminished ATCC 27853 reproductive viability but does not impair its metabolic activity. The arrow denotes the irradiation dose, which could reduce the reproductive viability to 0% while retained 63.33% ± 4.49% metabolic activity compared with the live cells (**C**). Results represent three independent experiments and are expressed as mean ± SD (n = 5).

Aliquots of *P. aeruginosa* ATCC 27853 were exposed to escalating X-ray irradiation or heat (65 °C), and the CFUs present from each aliquot were determined by plating on LB agar. As shown in Fig. 1A, an expected decrease in viability was detected with increasing heat duration or irradiation doses. A complete loss of replication viability of the bacteria was observed at a minimum dose of 3600 Gy, and the 45 min’s incubation at 65 °C inactivated the bacteria (Fig. 1). Likewise, the metabolic activity is also affected with heat or irradiation treatment. Of note, X-ray irradiation retained considerable metabolic activity of the inactivated bacteria, as indicated by the ability of irradiated ATCC 27853 to convert Alamar blue dye from blue to pink color. Metabolic activity of ATCC 27853 decreased as X-ray dosage increased, and the specific activity was 63.33% ± 4.49% when exposed to 3600Gy compared to that of the active live bacteria (100%). In contrast, heat-killed ATCC 27853 failed to cause the color change, which showed a complete loss of metabolic activity (Fig. 1). We then tested the *in vivo* proliferative activity of the irradiated bacteria, C57BL/6 mice were inoculated with 5** **×** **10^9^ CFU (intra-peritoneally, intra-nasally and subcutaneously) equivalent of either heat-killed (65 °C for 45 min) or irradiation inactivated *P. aeruginosa*, and no bacteria were detected in the main organs and blood of mice at one, three and five days post inoculation in both groups. We finally exposed the ATCC 27853 bacteria to 3600 Gy X-ray irradiation to manufacture an active but non-replicative whole cell vaccine.

We further immunized the mice with high dose vaccine (10^8^ CFUs) weekly by different routes, including subcutaneous, intra-nasal and intra-peritoneal method, and studied the potential long-term toxicity of the vaccine. We observed no adverse consequences in gross measures such as weight loss (Table S1.) or hematologic toxicities (Table S2, S3.). The immunized mice only exhibited purulent presentation at the injection site by the subcutaneous route, and had no clinical signs of weight loss, lethargy, piloerection, tremors, periorbital exudates, respiratory distress, or diarrhea. Furthermore, we investigated the pathologic changes of the immunized mice. One week after the fourth immunization, the immunized C57BL/6 mice, and unimmunized controls were all sacrificed, the main organ samples were taken out and applied for H&E staining to investigate the microscopic changes. The toxic pathologic changes in heart, liver, spleen, lung, and kidney were not detected by microscopic examination (data not shown). These results demonstrated that the vaccine did not cause obvious systemic toxicity.

### Protective efficacy of X-ray irradiated *P. aeruginosa* vaccination *in vivo*

**Figure 2:**
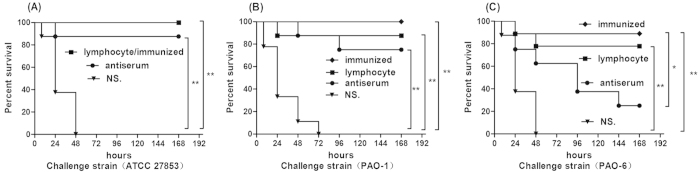
Evaluation of the vaccine induced protection against *P. aeruginosa* pneumonia *in vivo*. Immunized C57BL/6 mice received escalating doses of 10^8^, 5** × **10^8^, 10^9^, and 5** × **10^9 ^CFUs irradiated ATCC27853 cells intra-nasally every week. 1** × **10^7^ spleen lymphocytes or 300 μl serum were isolated from the immunized mice and transferred to normal mice 12 h before and after challenge, while controls received normal saline. Kaplan–Meier curves were plotted for mice of the above groups which were challenged by 5** × **10^6^ CFUs the parental strain ATCC 27853 (**A**), 5** × **10^6 ^CFUs homologous serotype PAO-1(**B**) and 1** × **10^7^ CFUs heterologous serotype PAO-6 (**C**), and monitored the seven days survival rates. Irradiated vaccine protected mice against intranasal challenge by virulent *P. aeruginosa*, serum transferred mice showed powerful anti-infectious effect against homologous strain (ATCC 27853 and PAO-1) while T lymphocytes adopter mice survived longer when challenged by the homologous and heterologous serotype strains (ATCC 27853, PAO-1, and PAO-6) than the controls. Results represent three independent experiments (n = 10, *p < 0.05, **p < 0.01).

We immunized mice by intra-nasal injection of irradiated vaccine or saline (unimmunized mice) once a week for four weeks, and then challenged the mice with *P. aeruginosa* strains. We found that the vaccine has significant protective effect against lethality in homologous serogroup PAO-1 (O2/O5) (p < 0.01) (Fig. 2A), the parent strain ATCC 27853 (O2/O5) (p < 0.01) (Fig. 2B) and heterologous serogroup PAO-6 bacteria (O6) (p < 0.01) (Fig. 2C), when compared with the controls after challenged by a relative high dose (five times of lethal dose 50) of the active live strains. Specifically, immunization lead to 100% protective effect in homologous serogroup compared with 87.5% in the heterologous serogroup PAO-6 challenge.

We further isolated sera and spleen lymphocytes from immunized mice and transferred them to the normal mice. Protective results were shown when mice that received spleen lymphocytes were challenged with the above three bacteria strains. However, the transferred sera only showed protective role for homologous serogroup PAO-1/ATCC 27853 infection (p < 0.01), and there was no significant protection after challenged by the heterologous serogroup PAO-6 (p > 0.05); only 22.5% anti-serum transferred mice survived seven days when infected by PAO-6 cells compared with 87.5% survival rate of the immunized group (Fig. 2).

### Opsonic killing activity of sera against *P. aeruginosa* in vitro

**Figure 3:**
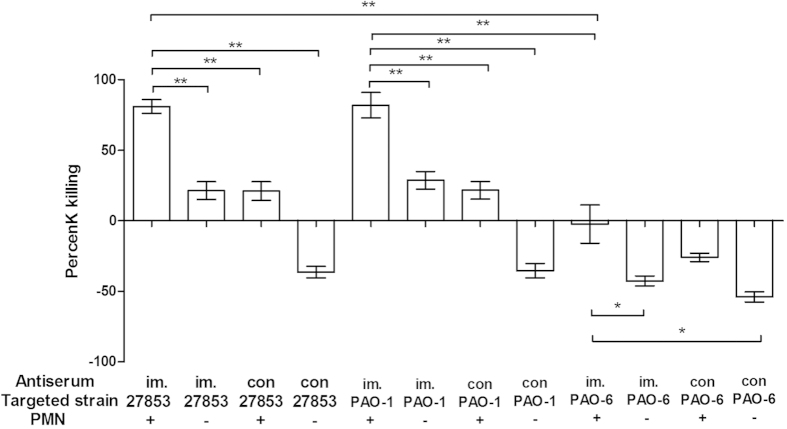
Opsonic killing activity elicited by the serum isolated from immunized mice against vaccine homologous strains (ATCC 27853, PAO-1) and vaccine heterologous strain (PAO-6) cells. Immunized group contained serum isolated from immunized mice, 1** × **10^6 ^PMN and 10^5^ CFUs heat-killed *P. aeruginosa* cells, while negative control group contained sera without PMNs. Serum isolated from immunized mice has higher killing activity against homologous strain (ATCC 27853 and PAO-1) cells than that of the controls (serum isolated from unimmunized mice). Results represent three independent experiments and are expressed as mean ± SD (n = 5, ANOVA, *p < 0.05, **p < 0.01).

The opsonic killing ability of anti-sera isolated from immunized mice were similar for either ATCC 27853 or PAO-1cells, and were about twice more effective than the activity of serum from unimmunized mice. The anti-serum demonstreated more effective killing ability against the homologous O2/O5 than the heterologous O6 strain bacterial cells (p < 0.05), and the immunized antiserum only has a tendency of proliferation inhibition to PAO-6 cells. As depicted in Fig. 3, there was minimal opsonic killing ability of strain ATCC 27853 (28.67 ± 2.51%) and PAO-1 (27.37 ± 3.17%) in the unimmunized mice serum, whereas effective killing was achieved by sera from immunized mice against the LPS-homologous ATCC 27853 (80.8% ± 2.02%) and PAO-1 cells (81.78% ± 3.24%). Surprisingly, although the replication of PAO-6 bacteria was not inhibited with the presence of anti-sera isolated from the immunized mice, there was a significantly higher killing ability when compared with that of serum isolated from control group mice (p < 0.05). All these results demonstrated that antibody is effective against homologous serotype *P. aeruginosa* cells proliferation, but only has partial protective role in heterologous serotype *P. aeruginosa* infection, which is in accordance with the survival protective results *in vivo*.

### CD4^+^ T lymphocytes response elicited by vaccine *in vitro* and *in vivo*

**Figure 4:**
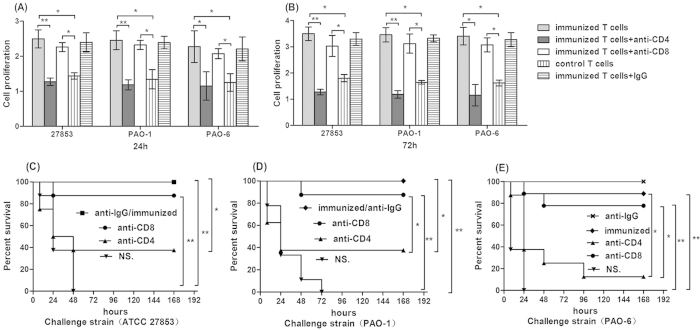
Role of CD4^+^ T lymphocytes in vaccine based protection against heterologous or homologous serotype strains. Proliferation of splenic T cells from vaccine immunized mice compared with that of unimmunized mice after the stimulation for 24 h (**A**) and 72 h (**B**) by heat-killed, whole bacterial cell antigen (ATCC 27853, PAO- and PAO-6). Immunized T cells have higher proliferation than the controls when stimulated by targeted cells at both time points, and the proliferation was significantly decreased by anti-CD4 antibody instead of CD8 antibody. Cells were pooled from three to five mice per group. Results were shown as mean ± SD (ANOVA, n = 3, *p < 0.05, **p < 0.01); Mice of control group were immunized with NS, and experimental groups were immunized with escalating doses of 10^8^, 5** × **10^8^, 10^9^ and 5** × **10^9^ CFUs irradiated ATCC 27853 cells every week. At the same time, mice were given either anti-CD4 monoclonal antibody (GK1.5), anti-CD8 monoclonal antibody (clone 2.43) or normal rat IgG to deplete CD4 and CD8 lymphoctyes, and then were challenged with 5** × **10^6^ CFUs the parental strain ATCC 27853 (**C**), 5** × **10^6^ CFUs homologous serotype PAO-1 (**D**) and 1** × **10^7^ CFUs heterologous serotype PAO-6 (**E**). Kaplan–Meier curves were plotted, and depletion of CD4^+^ T lymphocytes showed complete abrogation of the anti-infectious activity with the immunization, whereas depletion of CD8^+^ T lymphocytes did not affect the protective immunity (n = 10, *p < 0.05, **p < 0.01).

We isolated spleen T lymphocytes from immunized mice and co-cultured them with irradiated splenocytes as antigen presenting cells (APCs), heat-killed *P. aeruginosa* cells as antigens and measured T lymphocytes proliferation by CCK-8 test at 24 h and 72 h. As shown in Fig. 4, there was a higher proliferation level of immunized T cells to either ATCC 27853, PAO-1 or PAO-6 at both 24 h (Fig. 4A) and 72 h (Fig. 4B) when compared with those of T lymphocytes isolated from the controls (p < 0.05), which means vaccinated T lymphocytes could be stimulated by *P. aeruginosa* strains regardless of LPS serotypes. Besides, proliferation of the irradiated vaccine immune T lymphocytes was significantly inhibited by antibody to CD4 but not to CD8.

In order to further distinguish whether CD4^+^ T lymphocytes were the only immune cells involved in the anti-infection effect by X-ray irradiated vaccine *in vivo*, CD4^+^ or CD8^+^ T lymphocytes were depleted independently by corresponding antibodies as described above. We found that *in vivo* depletion of CD4^+^ T lymphocytes could completely abrogate the anti-infectious activity with the immunization. In the group of vaccine immunized group, the survival rate decreased to 37.5%, 37.5%, and 12.5% after depletion of CD4^+^ T lymphocytes when challenged with the strain ATCC27853, PAO-1, and PAO-6, respectively (Fig. 4). Furthermore, the survival rate was not decreased compared to the control group when challenged by ATCC 27853 (Fig. 4C), PAO-1 (Fig. 4D), since there existed effective killing ability of anti-serum against homologous serotype O2/O5. Depletion of CD8^+^ T lymphocytes showed that there was no relationship between CD8^+^ T lymphocytes and the anti-infectious effect. In addition, the treatment with normal rat IgG showed no improvement of the survival rate.

### Neutrophils recruitment induced by vaccination

**Figure 5:**
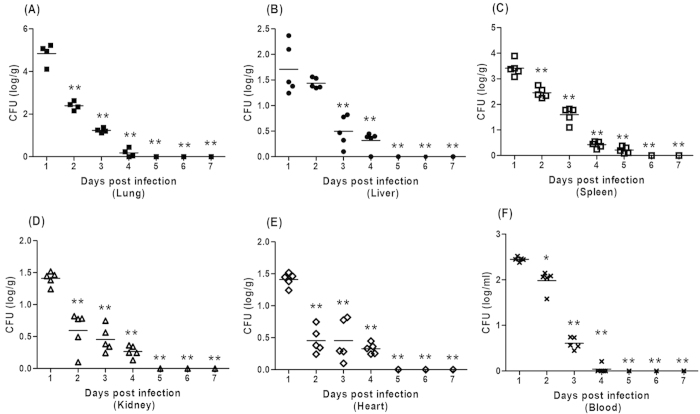
Immunization controls the bacteria spread in main organs and blood of mice after challenged by PAO-6. Immunized C57BL/6 mice received escalating doses of 10^8^, 5 × 10^8^, 10^9^, and 5 × 10^9^ CFUs irradiated ATCC27853 cells intra-nasally every week, and were challenged by 1 × 10^7^ CFUs PAO-6 cells. Main organs were collected and tested for bacteria number during the 7 days post challenge (ANOVA, n = 5, *p < 0.05, **p < 0.01 when compared with PAO-6 number at 24 hours).

Bacteria load is closely related with the survival status of infectious disease, so we test the bacteria number of the immunized mice post challenge to investigate the mechanisms involved in this protective effect of the vaccine against heterologous strain PAO-6 infection. It was interesting to note that the number of PAO-6 cells in the main organs and blood were decreasing within the seven days, and the mice finally clearly the spread of PAO-6 cells (Fig. 5). However, the control group mice almost died within twenty-four hours, and the corresponding bacteria number could not be detected.

**Figure 6:**
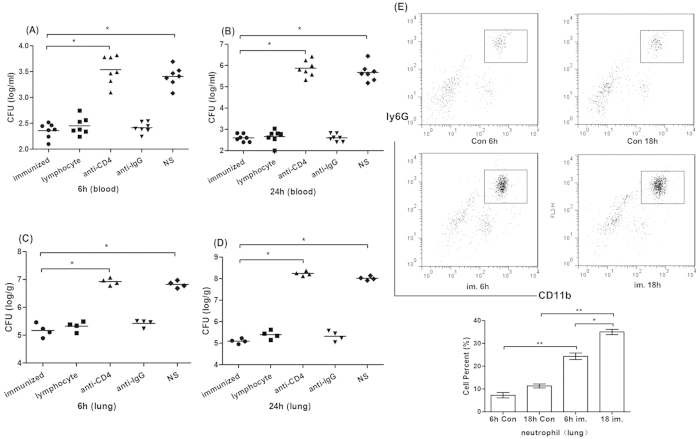
Immunization decreased bacteria load in the lung tissue and blood, and recruited more neutrophils early after heterologous serotype virulent PAO-6 challenge. Vaccine immunized mice, T lymphocytes transferred mice, CD4^+^ T lymphocytes depleted mice, IgG control mice and unimmunized mice were all challenged by 1** × **10^7^ CFUs PAO-6 cells. At 6 h (**A**), 18 h (**B**) after challenge the mice were anaesthetized, and the lung homogenate was collected and plated on agar plates (ANOVA, n = 7, *p < 0.05, **p < 0.01); At 6 h (**C**), 18 h (**D**) after challenge mice were anaesthetized, and the blood was collected and plated on agar plates (n = 4, ANOVA, *p < 0.05, **p < 0.01); (**E**) At 6h and 18h after PAO-6 challenge, lung tissue cells were recovered, firstly gated by CD45 and then stained by CD11b and Ly6G for neutrophil (CD45^+^ CD11b^+^ Ly6G^+^) identification. FACS data are presented with the means ± SD from all experiments for each group Results represent three independent experiments (n = 5, ANOVA, *p < 0.05, **p < 0.01).

When it comes to the CFUs in the lung tissue and blood of the PAO-6 infected mice early (at 6 h and 18 h) after challenge, as expected, the immunization resulted in the reduction of lung and blood bacterial load in comparison with that of the control group. We also explored the role of T lymphocytes in controlling the spread of PAO-6 cells, and found the adoptive transfer of lymphocytes significantly controlled the spread of PAO-6 cells, and the protective role could be reversed by CD4 antibody (Fig. 6A–D).

We tested the number of neutrophils (CD45^+^ CD11b^+^ ly6G^+^) recruited to the lung in different groups. As shown in Fig. 6E, there were significantly more neutrophils in the lung of immunized mice at 6 h and 18 h after challenged by PAO-6 when compared with that of the unimmunized controls (p < 0.05). Immunization could rapidly recruit neutrophils to the lung tissue since the number at 6 h is similar as that at 18 h post-infection, which explained the corresponding reduced bacteria load in lung and blood of infected mice early after infection.

### Th17 cells and IL-17 were activated by vaccine against LPS heterologous strain

**Figure 7:**
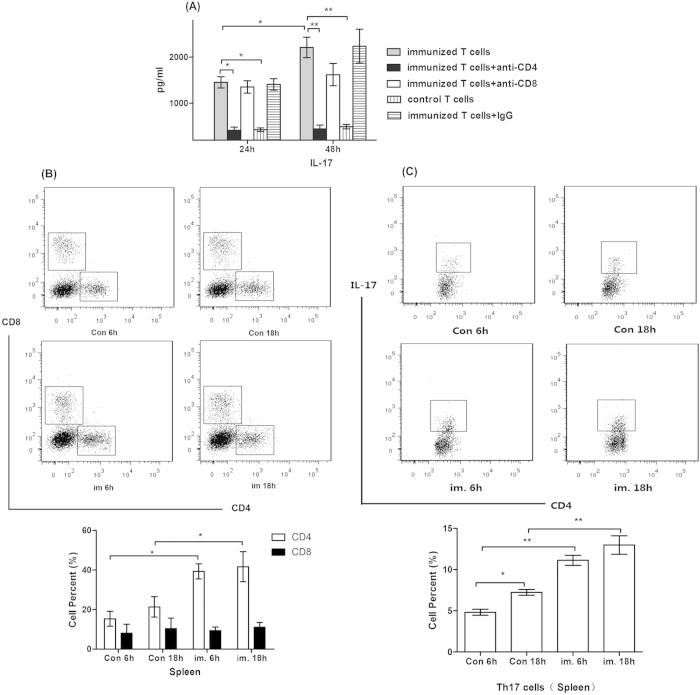
Nasal immunization with irradiated vaccine elicits Th17 cells responses. (**A**) IL-17 production by splenic T cells isolated from nasal immunized mice and then stimulated for 24 h and 48 h in the presence of heat killed whole bacterial cells of PA strain PAO-6 along with irradiated splenocytes. Immunized T cell secreted higher IL-17 than the controls, and IL-17 secretion decreases to baseline when splenic T cells are co-cultured with anti-CD4 monoclonal antibody (clone GK1.5, 1μg/well) at both time points (n = 3, ANOVA, *p < 0.05); (**B**) At 6h and 18h after PAO-6 challenge, spleen cells were recovered, gated by CD4 and CD8 for lymphocytes identification, and immunized mice has significantly higher CD4^+^ T cells compared with controls. FACS data are presented as means ± SD and represent three independent experiments (n = 5, ANOVA, *p < 0.05); (**C**) At 6 h and 18 h after PAO-6 challenge, spleen cells were recovered, gated by CD4 and IL-17 for lymphocytes identification, and immunized mice has significantly higher CD4^+^ IL-17^+^ Th17 cells compared with controls. FACS data are presented as means ± SD and represent three independent experiments (n = 5, ANOVA, *p < 0.05).

The levels of IL-17 in the supernatants of the immunized T cells were significantly higher than those of control T cells (p < 0.05). The presence of the anti-CD4 monoclonal antibody during co-culture returned the IL-17 levels as those of the control T cells, further indicating that CD4^+^ T cells are the predominant source of IL-17 in this system (Fig. 7A).

Mice challenged by PAO-6 were sacrificed at 6 h and 18 h to analyze the specific proliferation of T lymphocytes in the spleen *in vivo*. The data from Flow Cytometry analysis revealed that the number of CD4^+^ T cells in the group of vaccine-stimulated mice was significantly more than that in the controls at both time points (p < 0.05), while CD8^+^ T cells proliferation had no significant difference between each groups (p > 0.05) (Fig. 7B). The number of CD4^+^ IL-17^+^ Th17 cells in the vaccinated mice was significantly higher than in the controls at both time points (p < 0.05) (Fig. 7C), while the number of CD8^+^ IL-17^+^ Tc17 cells had no significant difference between each group (data not shown).

**Figure 8:**
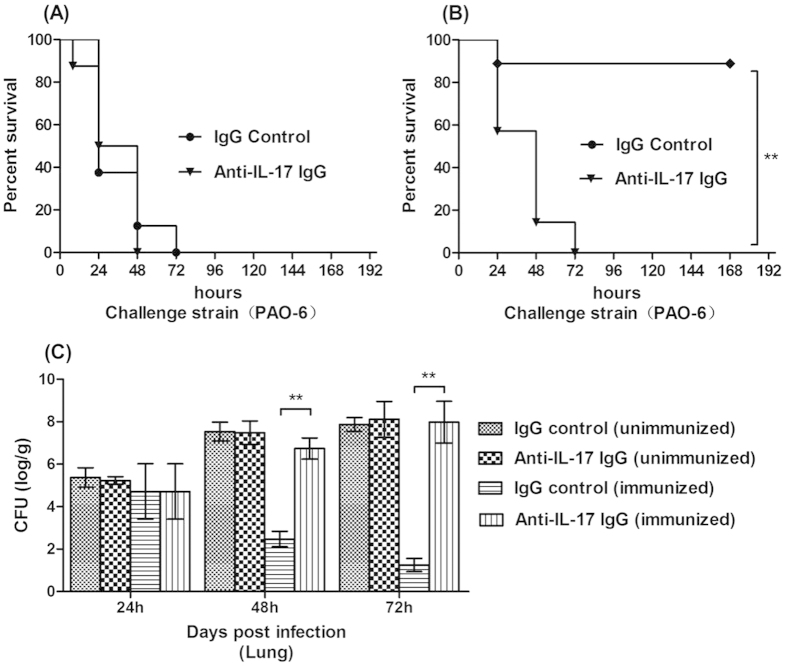
Neutralization of IL-17 diminishes vaccine induced protection against *P. aeruginosa* pneumonia. Survival of unimmunized (**A**) and immunized (**B**) mice after administration of IL-17 IgG or control IgG for consecutive 3 days prior to challenge with 5 × 10^7^CFUs PAO-6 (n = 8); (**C**) At 24, 48 and 72 hours post infection, the bacteria number of lung were measured in the immunized and control group mice which received either IL-17 IgG or control IgG (n = 5, ANOVA, *p < 0.05).

To further explore the role of IL-17 in the vaccine’s protective efficacy, we next determined the effects of neutralization of IL-17 prior to lung challenge with the PAO-6 in mice immunized intra-nasally with NS. Control (Fig. 8A) or the vaccine (Fig. 8B). As expected, there was no effect of IL-17 neutralization on survival condition following challenge of the unimmunized mice. However, there was significantly higher mortality in the immunized mice which received anti-IL-17 IgG compared with those given control IgG (Fig. 8A,B). Besides, we also tested the bacterial load of infected lung, and the immunized mice which received anti-IL-17 IgG also had higher level of PAO-6 number than the mice received control IgG, indicating that depletion of IL-17 abrogates vaccine induced protection against LPS heterologous strain challenge (Fig. 8C).

## Discussion

The wide array of virulence factors and the potential of *P. aeruginosa* to infect many different tissues have made it difficult to determine the main and most important microbial antigen targets for effective host immunity^19^. Thus the development of vaccines for *P. aeruginosa* has been hindered by the complexity of the organism’s pathogenesis as well as the host immune response^10^. Although multiple *P. aeruginosa* antigens have been studied as vaccine candidates, none of these has demonstrated broad protection against infection with multiple heterologous serogroup strains because they only targeted a single type of bacterial antigen^19^.

Both innate and adaptive immune responses work against bacterial infections^20^, *P. aeruginosa* is an extracellular pathogen, so humoral, mucosal or systemic opsonizing immunity is most effective preventing bacterial colonization and infection^21^,^22^. For most vaccines against *P. aeruginosa* infections, serum opsonic antibody directed against the LPS O antigen has been reported to be the most important immune effector. However, T lymphocyte responses have also been reported to mediate important protective immunity in individuals with *P. aeruginosa* infection^23^,^24^,^25^. Clinical studies revealed that effective resolution of infection or pathogens clearance is mediated by a combination of neutralizing antibody and inflammatory responses of activated macrophages, neutrophils and immune cells. Therefore, to prevent *P. aeruginosa* infections, an ideal vaccine should comprise a broad range of antigens to elicit both humoral and cellular immunic responses. In our study, significant protection against PAO-6 infection was achieved with the vaccine, and it was not associated with measurable antiserum opsonic killing activity. Our data suggested that the use of X-ray irradiated live-attenuated PA as a vaccine could induce broader, cross-protective cellular and humoral immunologic responses which are not limited to LPS O antigen, and thus expand the spectrum or the potency of protection.

We proved that X-ray irradiated *P. aeruginosa* strain ATCC 27853 vaccine could protect against acute lethal pneumonia caused by LPS-heterologous strain PAO-6. However, the antiserum had only good opsonic killing activity against homologous strain ATCC 27853 and PAO-1. Additionally, the protective effect via serum transfer was only realized when the mice were infected with the homologous strain. T lymphocytes were the effectors against heterologous strain PAO-6 infection, and *in vivo* CD4^+^ T cell depletion during immunization diminished the vaccine based protection against PAO-6 infection. However, the depletion of CD8 lymphocytes showed partial abrogation of the anti-infection activity when opsonophagocytic killing activity against the strain was absent. Our finding might explain the non effectiveness in reducing the incidence of *P. aeruginosa* infection of a passive administered antibody obtained from vaccinated volunteers from a recent clinical trial^26^. Our study also highlighted the fact that optimal adaptive immunity against the diverse PA strains requires both cellular and humoral effectors. Thus, cooperation of adaptive (CD4^+^ T cells and antibodies) and innate (neutrophil and macrophage) immunity can be optimized by vaccination with X-ray irradiated live-attenuated *P. aeruginosa* cells.

Bacterial metabolism has been demonstrated to play a major role in creating proper stimuli required for efficient triggering of protective responses^14^,^27^. A non-replicating but metabolically active vaccine would ensure that pathogen specific activity are maintained during the infectious process, including the presentation of bacterial antigens and triggering of host immune system. Traditional bacteria inactivation methods, via heat killing or chemical agents, denature the proteins and DNA thus impairing the pathogen’s replicating capability and metabolic activity^28^. Alternatively, irradiation provides an effective way to preserve metabolic ability when inactivating the bacteria, since it only causes fragmentation of DNA^17^,^29^. After irradiation, a large portion of the genome remains intact, so the bacteria have the potential to express genes in these segments and synthesize or secrete antigens or toxins^15^,^30^. Irradiation has been used as a method to prevent mammalian cell proliferation without inhibition of cellular activity^28^, the irradiated *Toxoplasma gondii* was reported to maintain morphology, metabolism, and cell invasion properties^31^. We showed that X-ray irradiated ATCC27853 cells retained the ability to transcribe proteins, maintain relevant targets for immune recognition, and diminish the corresponding replicative competence at the same time. Most importantly, our study suggested that metabolic activity is the key component mediating effective immunity in vaccine exploring. Future modifications to improve metabolic activity might enhance long term protective efficacy of vaccines.

As neutrophils are known to be essential mediators of host defense in the lung against *P. aeruginosa* infection and bacteria clearance, we supposed immunization may increase the number of neutrophils and thus control the spread and proliferation of PAO-6 bacteria. Of particular interest of our study is that the protective efficacy of vaccine against heterologous serotype PAO-6 infection coincided with a rapid reduction of bacterial load in lung tissue and blood as well as resulted in increased neutrophil numbers in lung as early as 6 h after intranasal challenge. Previous study reported that IL-17 from CD4^+^ T cells may induce enhanced production of antimicrobial peptides from lung epithelia or by chemokine production from fibroblasts and macrophages, leading to neutrophil recruitment and/or activation^32^, and the subsequent increased phagocytosis of bacteria and enhanced clearance of infection. These findings might be an explaination to the phenomenon that neutropenic patients have a higher incidence of Gram negative bacilli infection, and P. aeruginosais was reported as one of the leading Gram negative causative bacterial agent in neutropenic patients^32^,^33^.

IL-17 is known to stimulate neutrophil cells recruitment, so we supposed that the recently described Th17 subset of CD4 helper T cells, which secrete the neutrophil attracting cytokine IL-17, might play a role in the rapid recruitment of neutrophils to the lung. In our study, we observed high IL-17 level of immunized T cells after PAO-6 cells antigen stimulation, and high number of CD4^+^ IL-17^+^ Th17 cells in the spleen after challenge. Numerous studies have identified a protective role of IL-17 in immunity against various infections, including the infection of intracellular and extracellular bacteria^34^,^35^,^36^. Th17 cells have also been shown to play a critical role in the defense against *Klebsiella pneumonia*, *Pseudomonas aeruginosa* in murine models of airway infection, although the bacterial proteins recognized by the Th17 cells in those studies were not fully characterized^37^,^38^,^39^,^40^. In our own evaluations of live-attenuated *P. aeruginosa* vaccines, we found that Th17 cell was essential for LPS serogroup independent protection against pneumonia in the absence of opsonophagocytic antibody, it was also associated with rapid recruitment of neutrophils to the airways and the subsequent reduction of bacteria load. These findings indicated that CD4^+^ IL-17^+^ Th17 cells mediated immune response may be responsible for the anti-infectious activity afforded by the X-irradiated whole cell vaccine.

## Conclusion

This is an initial but significant step toward understanding the role of X-ray irradiated bacteria vaccine in *P. aeruginosa* infection. Irradiation of whole organisms proves to be a safe and immunogenic vaccine strategy in our preclinical study. In conclusion, our findings might provide a novel strategy of vaccine preparation against *P. aeruginosa* pneumonia, and this novel vaccine indeed obtained a better bacterial clearance effect. Furthermore, this strategy also saves the time and resources of identifying antigenic components for subunit vaccines preparation. The availability and rapidity of irradiation would expedite vaccine production and deployment during epidemic. Future studies will be required to establish this strategy as a viable preventable choice against human infection and determine the role of Th17 cells in the resistance to PA infection.

## Methods

### Animals and cell lines

All the protocols were performed in accordance with the approved guidelines. Female C57/BL6 mice (all 6–8 weeks of age) were obtained from the Beijing HFK Bioscience Co. Ltd. The mice were maintained at a twelve hours light and night cycle under specific pathogen free (SPF), temperature controlled conditions, and were fed with a standard laboratory diet during the entire experiment. All experimental procedures were approved by the Institutional Animal Care and Use Committee of Sichuan University.

Anti-CD4 mAb hybridoma cell (clone GK1.5, rat IgG), anti-CD8 mAb hybridoma cell (clone 2.43, rat IgG), and bacteria strains (ATCC 27853, PAO-1 and PAO-6) were all purchased from the American Type Culture Collection (Rockville, MD, USA). IL-17 antibody and the control IgG were purchased from Sigma (St. Louis, MO, USA). Hybridoma cell clone 2.43 was cultured in DMEM medium with 10% fetal bovine serum (FBS), and GK1.5 cultured in Iscove’s Modified Dulbecco’s (20% FBS) medium (Invitrogen). *P. aeruginosa* bacteria were all cultured with Luria–Bertani (LB) medium (Sigma-Aldrich, Shanghai, Trading Co. Ltd).

### Determination of bacterial replicate viability and metabolic activity

*Pseudomonas aeruginosa* ATCC27853 (serotype O2/O5) were grown in LB medium from a single colony for 16–18 h at 37 °C. The collected bacteria were adjusted to a concentration of 10^6^/ml and then exposed to various doses of X-ray at escalating intervals from 500 Gy to 6000 Gy by a RS2000 Biological X-ray irradiator (Rad Source Technologies, FL, USA) at 220 kV/40 mA. Similarly, the collected samples were incubated in a 65 °C water bath for 0, 20, 40, and 60 min and were kept at 4 °C until assayed. The replication viability of the bacteria were confirmed by plating serial dilutions on LB agar (BD Biosciences, Heidelberg, Germany), and the colony forming units (CFUs) on the bacterial culture plates were manually counted by a technician blinded to the experimental conditions after incubating for 24 hours.

The metabolic activity was assayed by Alamar Blue (BioSource International, Camarillo, CA), which incorporated a colorimetric growth indicator based on the detection of metabolic activity^41^,^42^. Briefly, the irradiated or heat incubated samples were firstly washed in normal saline (NS.) and then suspended in LB medium to the original concentration of 10^6^/ml. Then 100 μl irradiated suspensions were incubated together with 10 μl alamar blue dye for four hours in a 96-well plate. The metabolic activity was determined by detecting the absorbance at 600 nm and 570 nm, and subtracting OD_600_ from OD_570_^42^.

### Vaccine preparation

Cultured ATCC27853 cells were collected and suspended with NS. to a concentration of 10^10^/ml. One week before immunization, aliquots of the ATCC27853 cells were exposed to 3600 Gy irradiation. The inability of the irradiated bacteria to replicate was confirmed by plating on LB agar after incubating in a bacteria culture incubator for at least three days. The prepared aliquots of vaccine were then stored at −20 °C before immunization.

### Immunization, infection, and follow up

For the pneumonia model, mice were anesthetized and inoculated intra-nasally with irradiated ATCC27853 cells to further evaluate the protective effect generated by the vaccine^43^. Briefly, mice were immunized by placing 20 μl of the vaccine inoculums into each nasal (40 μl per mouse totally). Escalating doses of 1** × **10^8^, 5** × **10^8^, 10^9^, and 5** × **10^9^ CFUs were administered at weekly intervals, while the mice in the control group received equal volume of NS.

One week after the fourth immunization, C57/BL6 mice in both groups were intra-nasally challenged with 5** × **10^6^ CFUs equivalents of active ATCC27853, PAO-1 (serotype O2/O5) or 1** × **10^7^ CFUs PAO-6 (serotype O6). Then, the mice were monitored for seven days’ survival rates and bacteria loads after the infection.

In different sets of experiments further explained in the results part, the mice were sacrificed at predetermined time points after immunization or challenge, samples (serum, blood, or organs) were harvested for cytokine levels, bacteria load measurements, lymphocytes/serum extraction or determination for the proportion of neutrophils or Th17 cells by Flow Cytometry. The decision to sacrifice mice was made before the experiment was started.

### Adoptive transfer or depletion of spleen lymphocytes (serum) *in vivo*

Preparation of spleen lymphocytes and serum was performed according to the method described previously^44^. Briefly, the immunized mice were sacrificed a week after the fourth immunization, blood samples were collected to coagulate and then centrifuged at 1000 g for 20 min to isolate the serum. Mice spleen was harvested at a sterilized condition, cell suspensions were generated through a 70 μm nylon mesh filter (BD Biosciences), and lymphocytes were enriched by specific separation medium and density gradient centrifugation.

For Adoptive transfer of antiserum or lymphocytes experiment, 1** × **10^7^ lymphocytes or 300 μl serum were adoptively transferred intravenously 12 h before and after C57BL/6 mice were challenged with live virulent ATCC 27853, PAO-1, or PAO-6 cells. For lymphocytes depletion experiment, immune cell subsets were depleted as described previously^44^. Mice were injected intraperitoneally with 500 μg monoclonal antibodies against CD8, CD4, or the isotype control IgG antibody one day before the first immunization, and then twice a week for four weeks until the immunization accomplished. Then the immunized mice, CD4^+^ T lymphocytes depleted mice, CD8^+^ T lymphocytes depleted mice, and the unimmunized mice were all challenged with live ATCC 27853, PAO-1, or PAO-6 cells, and then were monitored for seven days survival rates after intra-nasal infection. Similarly, IL-17 depletion studies were done using anti-IL-17 IgG (1 mg i.p.) or control IgG for consecutive 3 days before the mice were challenged by PAO-6 cells.

### Opsonophagocytic assays

Standard methods were employed to test the antibody dependent opsonophagocytic killing ability of antiserum isolated from the immunized mice^45^. Briefly, 2** × **10^6^ cells polymorphonuclear leukocytes (PMNs) from human volunteers, 5** × **10^5^ CFUs PA targeted strain (ATCC 27853, PAO-1, or PAO-6), and 200 μl anti-sera were mixed in a sterile micro-centrifuge tube and incubated for 4 h at 220 rpm/min. The anti-sera were collected and pooled after the fourth immunization as mentioned above. Control group contained serum isolated from the unimmunized mice, and tubes with serum isolated from unimmunized mice but without PMNs served as additional negative controls to help distinguish killing from agglutination^46^.

After incubation, each well was subjected to serial log-fold dilutions to determine the bacterial CFUs, and the decreased percent of bacterial CFUs in the experimental tube compared to that in tubes incubated with unimmunized sera was calculated as the opsonophagocytic activity. Assays were performed in three times for each sample. Under routine conditions, killing of above 50% is considered biologically significant and we can classify the serum as positive for opsonophagocytic killing activity.

### T cell proliferation and cytokine measurements

For proliferation experiments^47^, each well of a 96-well plate was seeded with 1** × **10^5^ T cells isolated from immunized mice, 1** × **10^5^ irradiated (1500 rad) splenocytes isolated from normal mice as antigen presenting cells (APCs), and 1** × **10^6^ heat-killed bacteria (ATCC 27853, PAO-1, or PAO-6) as antigen, while additional groups contained 1 μg per well anti-CD4, anti-CD8, or rat IgG isotype (BD Biosciences) antibody to identify the specific proliferation ability of different group lymphocytes. The well of control group contained T cells isolated from the unimmunized mice, and the cells were all cultured by RPMI 1640 containing 10% heat-inactivated FBS. The killed bacteria were verified by the absence of growth on LB agar.

At 24 h and 72 h after incubation, T lymphocyte proliferation in above different groups was assessed using Cell Counting Kit-8 (CCK-8) assay according to the manufacturer’s protocol. At the same time, the supernatants in each well were collected for testing IL-17 cytokine levels using the commercial mice ELISA kit (Sigma-Aldrich, Shanghai, China).

### Quantification of bacterial counts in blood and main organs

The immunized and control group mice were sacrificed by dislocation of lumber bar at predetermined time after the induction of intranasal infection by PAO-6 cells. Blood (bled from the retro-orbital sinuses) and organ tissue homogenate were harvested under sterile conditions and subjected to serial log-fold dilutions using sterilized NS. The dilutions from all samples were then plated onto sheep blood agar plates (BD Biosciences, Heidelberg, Germany). After overnight incubation, the CFUs of the bacterial culture plates were manually counted by a technician blinded to the experimental conditions. Data were expressed as CFUs per mL blood or per g of lung tissue.

### Flow cytometry

The immunized and control animals were sacrificed by dislocation of lumber bar at 6 h and 18 h after challenged with PAO-6 cells. Spleen and lung tissue single cell suspensions were harvested by method described previously^48^. Lung cells were incubated for 30 min on ice with 1 μL of relevant Abs (CD45-PE, CD11b-APC, and ly6G-FITC) or matched isotype control Abs for detection of neutrophils. Intracellular cytokine staining was performed using a kit from BD Biosciences according to the manufacturers’ instructions. Spleen cells of immunized or control mice were stained with CD4-APC or CD8-Alex Flour 488 mice antibody, followed by fixation and permeabilization and then were stained with PE-labeled IL-17 antibody. Antibodies and appropriate isotype controls were also obtained from BD Biosciences. Flow cytometry data were acquired by a FACS Calibur flow cytometer (BD Biosciences) and analyzed with FLOW JO software 7.6 (Tree Star Inc., Ashland, OR).

### Statistical analysis

All data were analyzed using GRAPHPAD PRISM software (GraphPad, San Diego, CA). Data were analyzed using ANOVA (multiple groups), and multiple comparisons between the groups were performed using Newman–Keuls method after ANOVA. Survival data were plotted using Kaplan–Meier curves and analyzed by the log-rank test. For measurements of bacterial CFUs, groups were compared using a nonparametric Mann–Whitney U-test. p < 0.05 was considered to be statistically significant for all experiments. All values were presented as the mean ±SD, with the exception of bacterial counts, for which median values were designated.

## Additional Information

**How to cite this article**: Li, Y. *et al.* X-ray Irradiated Vaccine Confers protection against Pneumonia caused by *Pseudomonas Aeruginosa*. *Sci. Rep.* **6**, 18823; doi: 10.1038/srep18823 (2016).

## Supplementary Material

Supplementary Table 1
