# Supplementary material for: X-ray Irradiated Vaccine Confers protection against Pneumonia caused by Pseudomonas Aeruginosa
Source: Sci Rep. 2016 Feb 16;6:18823. doi: 10.1038/srep18823 (PMC4754647; doi:10.1038/srep18823)
Supplement: Supplementary Table 1 [file srep18823-s1.doc]

**X-ray Irradiated Vaccine Confer protection against Pneumonia caused by *Pseudomonas Aeruginosa***

Yanyan Li ^1,2^, Zhenling Wang ^2^, Xiaoxiao Liu ^2^, Jianying Tang ^2^, Bin Peng^2,3^, Yuquan Wei ^2^*

1. Department of Radiation Oncology, Fudan University Shanghai Cancer Center, Fudan University, Shanghai, China.

2. State Key Labortary of Biotherapy and Cancer Center, West China Hospital, Sichuan University, and Collaborative Innovation Center of Biotherapy, Cheng Du, China.

3. Department of Oncology, Third Xiangya Hospital, Central South University, Changsha, China.

Yanyan Li, Zhenling Wang, and Xiaoxiao Liu contributed equally to the work.

Corresponding Author:

Wei Yuquan, State Key Labortary of Biotherapy and Cancer Center, West China Hospital, Sichuan University, and Collaborative Innovation Center of Biotherapy, Cheng Du, 610041, China.
Tel./fax: +86-28-85164063

E-mail: [yqwei@scu.edu.cn](mailto:yqwei@scu.edu.cn)

**Table S1. Weight Change of Mice at Different Times Post Immunization (g)**

| Group/week | Before im. | Third week | Sixth week | Ninth week |
| --- | --- | --- | --- | --- |
| Subcutanous | 19.20±1.23 | 21.91±2.47 | 28.94±1.94 | 32.02±2.13 |
| Control 1 | 18.52±2.03 | 21.81±2.11 | 27.28±2.01 | 33.32±1.34 |
| Intra-peritoneal | 20.08±0.90 | 21.41±1.81 | 28.81±0.90 | 32.12±1.12 |
| Control 2 | 19.23±1.57 | 21.73±1.16 | 27.78±0.45 | 33.41±1.05 |
| Intra-nasal | 19.31±1.33 | 20.12±1.21 | 24.90±1.67 | 31.14±1.24 |
| Control 3 | 20.01±0.70 | 22.01±1.13 | 26.34±1.43 | 32.45±2.33 |

**Table S2. Blood Test of the Immunized Mice** (Units)

|  | RBC  (1012/L) | HGB  (g/L) | WBC  (109/L) | HCT  (%) | PLT  (109/L) | MCV  (fL) |
| --- | --- | --- | --- | --- | --- | --- |
| Immunized | 8.6±0.8 | 118.1±10 | 3.0±0.8 | 49.2±3.9 | 775+110.7 | 49+1.8 |
| Control | 8.4±0.6 | 121±7.5 | 2.8±0.7 | 44.2±2.5 | 667+76.3 | 47.5+2 |

RBC：red blood cells；HGB：hemoglobin；WBC：white blood cell；PLT：platelet；

HCT：Red blood cell specific volume；MCV：erythrocyte mean corpuscular volume

**Table S3. Liver and Kidney Function Test of the Immunized Mice** (Units)

|  | ALT  (U/L) | AST  (U/L) | TP  (g/L) | ALB  (g/L) | UREA  (mmol/L) | UA  (mg/dL) |
| --- | --- | --- | --- | --- | --- | --- |
| Immunized | 67.2±9.2 | 88.1±10.3 | 58.1±10.9 | 32.8±8.0 | 8.06+1.99 | 53.8+4.9 |
| Control | 65.2±7.6 | 86.4±7.5 | 57.8±5.7 | 34.6±2.7 | 7.92+2.05 | 57.3+6.2 |

ALT：Alanine aminotransferase；AST：Aspartate transaminase；TP：total protein；

ALB：albumin；UA：Uric acid
